# Surfactant protein-D is an independent predictor of all-cause mortality in men with peripheral artery disease diagnosed by population-based screening

**DOI:** 10.3389/fcvm.2025.1534779

**Published:** 2025-05-20

**Authors:** Kimmie B. Christensen, Lasse M. Obel, Jes S. Lindholt, Grith L. Sorensen

**Affiliations:** ^1^ Department of Molecular Medicine, Faculty of Health Sciences, University of Southern Denmark, Odense, Denmark; ^2^ Department of Cardiothoracic and Vascular Surgery, Odense University Hospital, Odense, Denmark

**Keywords:** peripheral artery disease, surfactant protein-D, biomarker, cardiovascular disease, atherosclerosis

## Abstract

**Introduction:**

Peripheral artery disease (PAD) is a common condition caused by atherosclerosis, which leads to reduced blood flow to the limbs. PAD is associated with major adverse cardiovascular events (MACE) and major adverse limb events (MALE). Surfactant protein-D (SP-D) is a defense lectin expressed in the lungs and vasculature and has been previously associated with PAD.

**Methods:**

We aimed to examine the prognostic value of plasma SP-D in relation to MACE, MALE, and all-cause mortality in 913 men with PAD diagnosed through population-based screening. The cohort was divided into low plasma SP-D (<420.4 ng/ml) and high SP-D (≥420.4 ng/ml) based on the 95th percentile of baseline measurements. The data were analyzed using univariate and multivariate Cox regression analyses.

**Results:**

SP-D was not associated with MACE or MALE. All-cause mortality was significantly increased in the high SP-D group compared with the low SP-D group (31.1% vs. 14.9%, *p* = 0.003), with an adjusted hazard ratio of 2.40 (1.36–4.24), *p* = 0.003, over a mean follow-up period of 5.2 ± 1 years.

**Discussion:**

SP-D is not associated with MALE and MACE but is an independent predictor of all-cause mortality in men with PAD diagnosed through population-based screening.

## 1 Introduction

Peripheral artery disease (PAD) refers to the narrowing or blockage of peripheral arteries due to atherosclerosis, leading to a range of clinical symptoms and complications. This condition spans from asymptomatic cases, characterized by a compromised ankle–brachial index, to varying degrees of intermittent claudication, severe night and rest pain indicative of critical limb ischemia, and the presence of non-healing ulcers and tissue loss. PAD shares the same risk factors as atherosclerosis, such as advanced age, smoking, and diabetes. PAD is a common disease with a global prevalence of 5.6% and is relatively more prevalent in high-income countries (1). PAD is diagnosed by measuring the ankle–brachial index (ABI), with an ABI of <0.9 or >1.4 indicating PAD (2).

The risk of cardiovascular disease (CVD) mortality in PAD patients is almost doubled when age-adjusted and increases with the severity of PAD (3). Hence, PAD is highly associated with major adverse cardiovascular events (MACE), such as stroke, myocardial infarction, and cardiovascular death (3, 4). PAD is also highly associated with an increased risk of major adverse limb events (MALE), such as acute and chronic limb ischemia, amputation, and arterial revascularization (5). Despite its high prevalence and strong association with other comorbidities and death, PAD remains an underdiagnosed and undertreated disease (6), and prognostic PAD biomarkers are highly warranted.

Surfactant protein-D (SP-D) is a soluble host defense lectin involved in innate immunity and is highly expressed in the lungs (7). It is also found at other sites such as in the cardiovascular system (7–10). Constitutional circulatory SP-D levels are found to be highly genetically determined, yet also highly variable (11).

The relation between the highest circulatory SP-D levels and mortality has been recognized for many years (12, 13). Bronchial and circulatory SP-D are induced with pulmonary disease and have been associated with mortality in several studies of pulmonary disease (14–19). However, while SP-D is primarily synthesized in the lungs, the source of circulatory SP-D is not fully clarified. Lung spillover might be the primary contributor to circulatory SP-D, but secretion of SP-D directly from the cells of the arterial wall also has the potential to contribute (8). Important for the rationale for studying SP-D as a CVD biomarker, Hill et al. (20) in 2011 demonstrated that circulatory SP-D is not only associated with mortality in lung disease patients but also in patients suffering from cardiovascular disease. In line with this notion, circulatory SP-D was independently and positively associated with both carotid artery intima–media thickness and severe coronary artery calcification (21), while not in subclinical atherosclerosis (22). An additional rationale for studying SP-D as a biomarker in CVD is that SP-D is recognized as a molecular driver of atherogenesis through studies of gene deficiency (9, 23).

The discovery that circulatory SP-D had potential as a prognostic biomarker in CVD led Otaki et al. (24) to examine if SP-D was associated with the clinical outcome of 364 PAD patients. They found that patients with high circulatory SP-D levels had an increased risk of MACE and MACLE (MALE + MACE) and that SP-D could serve as an independent predictor of these events (24). In the present study, we set out to analyze a new PAD cohort to see if we could replicate such findings. The present study aimed to examine the value of plasma SP-D as a prognostic biomarker of PAD degree, MACE, MALE, MACLE, and all-cause mortality in a cohort of 913 men with screening-diagnosed PAD.

## 2 Materials and methods

### 2.1 Patients with PAD

The study was conducted according to the guidelines of the Declaration of Helsinki and approved on 23 March 2008 by the Regional Scientific Ethics Committee of the Region of Central Denmark (M20080028). The patients included in this study were part of the Viborg Vascular (VIVA) screening trial of 65–74-year-old men in the Central Region of Denmark (25). A total of 18,749 participants were enrolled in the study and screened for PAD and abdominal aortic aneurysms between October 2008 and January 2011.

In the VIVA trial, the upper limb with the highest blood pressure (BP) was used as a reference. Brachial and ankle pressures were recorded simultaneously, with ankle pressure calculated as the mean of two pedal artery measurements, repeated on the opposite leg (26). Blood pressure values were averaged from the last two readings. PAD was diagnosed with an ankle–brachial index (ABI) of ≤0.9 or ≥1.4. In this study, PAD patients were divided into groups based on the degree of PAD severity: PAD degree 1 (0.75 < ABI < 0.89 or 1.40 < ABI < 1.60), PAD degree 2 (0.5 < ABI < 0.75 or 1.6 < ABI < 1.8), and PAD degree 3 (ABI < 0.5 or >1.8). Examiners, trained using a validated protocol, conducted independent measurements if the variation between assessors remained below 15%. The patients were classified as having symptomatic PAD if they experienced pain while walking that subsided immediately upon stopping; otherwise, they were categorized as having asymptomatic PAD.

PAD was found in 2,043 of the participants, equal to 10.9% (25). Of these, plasma samples from 1,160 male patients were available. Patient samples were excluded if patients were using glucocorticoid inhalation or tablets (*n* = 101), were misclassified as PAD patients (*n* = 64) (0.9 < ABI < 1.4), or did not have a complete medical record (*n* = 98). In total, 913 male patients with PAD were included in the final study. A thorough medical history was recorded for all patients, including the use of statins, antihypertensive drugs, and aspirin. Hypertension was defined as blood pressure (BP) exceeding 160/100 mmHg or the use of antihypertensive drugs. Diabetes mellitus (DM) was defined by a history of diabetes or the use of antidiabetic drugs or insulin. The patients were characterized as current smokers, previous smokers, and non-smokers, based on the medical interview at the time of inclusion in the VIVA trial. Smoking status during follow-up and newly diagnosed cases of chronic obstructive lung disease or changes in pharmacological treatment were not collected; these relied on data collected at baseline. MALE and MACE were identified in the National Danish Patient Registry (27); please see Supplementary Table S1 for definitions. All-cause mortality data were obtained from the Danish National CPR registry (28).

### 2.2 Detection of plasma SP-D by the AlphaLISA technique

The AlphaLISA technique (PerkinElmer) was used for measuring SP-D in the samples. This bead-based ELISA-like method does not require washing steps and is characterized by high sensitivity, a wide dynamic range, and robust performance. A biotinylated antibody to the analyte binds to the streptavidin-coated donor beads, while a second antibody to the analyte is directly conjugated to AlphaLISA acceptor beads. In the presence of the analyte, the two beads come into proximity. Excitation of the donor beads at 680 nm generates singlet oxygen molecules that trigger a series of chemical reactions in the acceptor beads, resulting in a sharp peak of light emission at 615 nm.

Briefly, a monoclonal anti-SP-D antibody (HG-HYB246-05) was conjugated to AlphaLISA acceptor beads (PerkinElmer) at a concentration of 0.6 mg antibody/mg acceptor beads following the manufacturer's instructions. Monoclonal anti-SP-D antibody (HG-HYB246-06) was modified by labeling with biotin (N-hydroxysuccinimide-biotin) (Sigma-Aldrich H1759) to permit binding to AlphaLISA streptavidin-coated donor beads. The AlphaLISA procedure was performed using 384-well microtiter plates (white opaque OptiPlate from PerkinElmer, #6007290) containing 5 µl of diluted plasma and 20 ul of a mix with biotinylated HG-HYB246-06 diluted 1:50 in AlphaLISA binding buffer (500 mM Tris pH 7.4, 1% BSA, 0.15 Triton X-100, 50 mM CaCl_2_) and HYB246-05 conjugated to acceptor beads diluted 1:20 in AlphaLISA binding buffer. The plate was incubated at room temperature for 1 h in the dark.

Then, 25 µl of streptavidin donor beads diluted 1:62.5 in AlphaLISA binding buffer was added, and the plate was incubated at room temperature in the dark for another 30 min, after which it was read on an EnSpire reader (PerkinElmer) using the AlphaScreen protocol. The experiments were performed in duplicate except for the standards, which were performed in quadruplicates. Duplicate sample variance was accepted if it was ≤10%. Standards were prepared from a stock solution of 1 mg/ml of recombinant human (rh)SP-D diluted in fetal bovine serum (FBS). Standards included serial dilutions from 200 ng/ml to 1.56 ng/ml. Quality controls were prepared from FBS spiked with rhSP-D in four levels, aliquoted, frozen, and then included on each plate. The inter-assay coefficient of variation (CV) of <10% (9.2%, 5.2%, 6.2%, and 4.6%, respectively, for the four control samples) was calculated using measurement of the four control samples included in the independent consecutive analyses of eight to nine vidual 384-well microtiter plates. All samples were measured in duplicates and the intra-assay CVs were calculated for all these samples individually. The sample measurement was repeated if this intra-assay CV was above 10%.

### 2.3 Statistical methods

**Figure 1:**
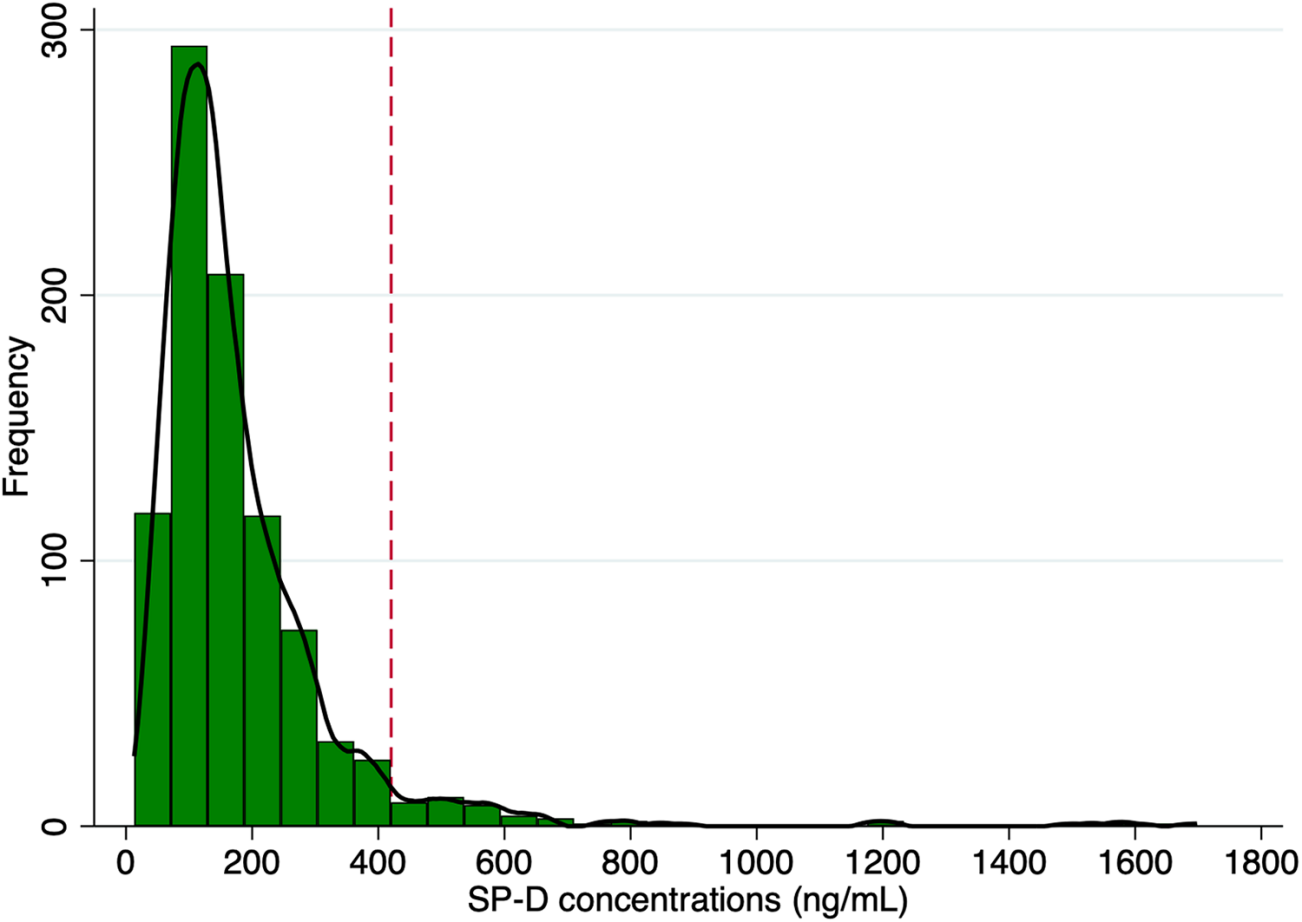
Histogram of the distribution of plasma SP-D levels. Frequencies of levels of SP-D concentrations in 913 men with screening-diagnosed peripheral artery disease. The black solid line represents the Kernel density estimates. The red dashed line represents the 95th percentile (420.4 ng/ml).

The distribution of plasma SP-D concentrations was visually evaluated through probability plots and appeared to follow a close-to-normal distribution but with a long right-skewed tail (Figure 1). SP-D concentrations were then divided into a “high SP-D” group using the 95th percentile (SP-D ≥ 420.4 ng/ml) and a “low SP-D” group (SP-D < 420.4 ng/ml). The continuous variables were expressed as means with 95% confidence intervals (95% CI). The continuous data were checked for normal distribution with Shapiro–Wilk tests and Q–Q plots. Differences between groups were compared with Student's *t*-test or the non-parametric Mann–Whitney *U*-test depending on normality. The rank-sum test was used to compare overall SP-D levels between symptomatic and non-symptomatic PAD patients, while the Kruskal–Wallis test was applied to assess differences in SP-D levels across PAD severity categories. The categorical variables were presented as proportions (*n*) and compared with the chi-square test. All-cause mortality frequencies were presented in a Kaplan–Meier plot stratified by the “high SP-D” group and the “low SP-D” group. The difference in survival rates was tested with the log-rank test. Univariate Cox proportional hazard analyses were used to identify potential predictors of all-cause mortality, MALE, MACE, and MACLE based on classical CVD risk factors, respectively. Potential predictors with *α* levels of <0.10 from univariate testing were subsequently adjusted for in multivariable Cox proportional regression analyses. A *p-*value of <0.05 was considered statistically significant. The receiver operating characteristic (ROC) curve was used to estimate the performance of a “high SP-D” as a marker of increased mortality at the chosen threshold. Stata/IC 16.1 was used for statistical calculations.

## 3 Results

### 3.1 Baseline characteristics of PAD patients

The distribution of plasma SP-D levels in patients with PAD is shown in Figure 1. The baseline characteristics of patients with PAD, divided into low plasma SP-D (<420.4 ng/ml) and high SP-D (≥420.4 ng/ml), are shown in Table 1. The low SP-D group included 868 (95.1%) male patients, and the high SP-D group included 45 (4.9%) male patients.

**Table 1:** Baseline characteristics and outcomes of PAD patients divided by the 95th percentile of SP-D into low SP-D (<420.4 ng/ml) and high SP-D (≥420.4 ng/ml).

Groups	Total *N*	Low SP-D (*n* = 868)	High SP-D (*n* = 45)	*p*-value
SP-D, ng/ml	913	13–420.4	420.4–1,698	
Age, years	913	69.5 [69.3–69.7]	69.5 [68.7–70.3]	0.99
BMI, kg/m^2^	913	26.6 [26.3–26.8]	25.6 [24.3–26.9]	0.13
Smoking status	912	40.7 (359)	43.5 (20)	0.71
Never, % (*n*)	112	12.2 (106)	13.3 (6)	0.98
Former, % (*n*)	428	46.9 (407)	46.7 (21)	
Current smokers, % (*n*)	372	40.8 (354)	40.0 (18)	
COPD	913	23 (2.7)	<3	0.47
Diastolic BP, mmHg	909	83.0 [82.2–83.7]	80.0 [76.0–84.0]	0.09
Systolic BP, mmHg	910	159.1 [157.6–160.6]	151.4 [145.1–157.7]	**0**.**024**
PAD Degree	913			
1	311	33.8 (293)	40.0 (18)	0.16
2	407	45.3 (393)	31.1 (14)	
3	195	21.0 (182)	28.9 (13)	
Hypertension, % (*n*)	894	83.4 (708)	86.7 (39)	0.56
DM, % (*n*)	911	17.9 (155)	26.7 (12)	0.14
Aspirin, % (*n*)	898	57.3 (489)	75.6 (34)	**0**.**016**
ACE inhibitors, % (*n*)	858	32.4 (264)	43.2 (19)	0.14
Beta-blockers, % (*n*)	866	28.6 (235)	56.8 (25)	**<0**.**001**
Calcium blockers, % (*n*)	863	30.9 (253)	34.1 (15)	0.66
Beta-agonists, % (*n*)	836	2.0 (16)	0.0 (0)	0.35
Statins, % (*n*)	895	58.0 (493)	60.0 (27)	0.79
Warfarin, % (*n*)	841	8.8 (70)	9.3 (4)	0.91
Previous CVD, % (*n*)	913	21.2 (184)	37.8 (17)	**0**.**009**
Outcomes				
MALE, % (*n*)	913	11.6 (101)	15.6 (7)	0.43
MACE, % (*n*)	913	21.4 (186)	24.4 (11)	0.63
All-cause mortality, % (*n*)	913	14.9 (129)	31.1 (14)	**0**.**003**

Data are presented as means [95% confidence intervals] for continuous variables and percentages (numbers) for dichotomous variables, respectively. *p*-values are derived using the Wilcoxon rank-sum test or the Student's *t*-test for continuous variables, as appropriate, and the chi-squared test for dichotomous variables, respectively. A *p*-value of <0.05 is written in bold, indicating a significant difference between the low and high SP-D groups. ACE, angiotensin-converting enzyme; BP, blood pressure; BMI, body mass index; CVD, cardiovascular disease; DM, diabetes mellitus; MACE, major adverse cardiovascular event; MALE, major adverse limb event; PAD, peripheral artery disease; SP-D, surfactant protein-D.

Among the 913 included PAD patients, symptom data were missing for 10 individuals, leaving 903 individuals for analyses of SP-D levels in symptomatic vs. asymptomatic patients. Among symptomatic PAD patients, 4.9% (16/325) were in the high SP-D group, compared with 5.0% (29/578) in the asymptomatic group (*p* = 0.95). The overall median SP-D levels were 142 (IQR: 100–201) in symptomatic PAD and 140 (IQR: 95–212) in asymptomatic PAD (*p* = 0.60). No significant differences in SP-D levels were observed when stratifying by PAD severity and symptom status (*p* = 0.59), as shown in Supplementary Table S2.

For an overview of the distribution of mean ABI measurements at baseline, please see Supplementary Figure S1.

Analysis of clinical variables between groups showed a significant difference in systolic blood pressure, use of aspirin and beta-blockers, and previous CVD events. There were no significant differences between age, BMI, smoking status, diastolic blood pressure, prevalence of hypertension, chronic pulmonary disease, diabetes, and use of various other medications. The unadjusted baseline characteristics of patients divided into low and high SP-D did not show any significant differences in MALE or MACE events, but a significantly increased proportion of patients with high SP-D died during follow-up (Table 1).

### 3.2 Clinical outcomes in PAD patients

During the follow-up period of mean 5.2 ± 1.0 years, there were 108 (11.8%) incidents of MALE, 197 (21.6%) incidents of MACE, and 143 (15.7%) incidents of death. Cox regression analysis showed that there was no significant difference in hazard ratio of MALE [adjusted HR = 1.32, 95% CI (0.60–2.87), *p* = 0.49], MACE [adjusted HR = 1.18, 95% CI (0.64–2.17), *p* = 0.60], or MACLE [HR = 1.01, 95% CI (0.55–1.87), *p* = 0.97] between the high and low SP-D groups (Supplementary Tables S1, S2, and S3, respectively). Hypertension, use of beta-agonists, and increasing PAD degrees significantly and independently increased the risk for MALE, while increasing age, hypertension, diabetes mellitus, previous CVD events, and PAD degree increased the risk for MACE. Similar results were observed for the combined MACLE outcome.

**Figure 2:**
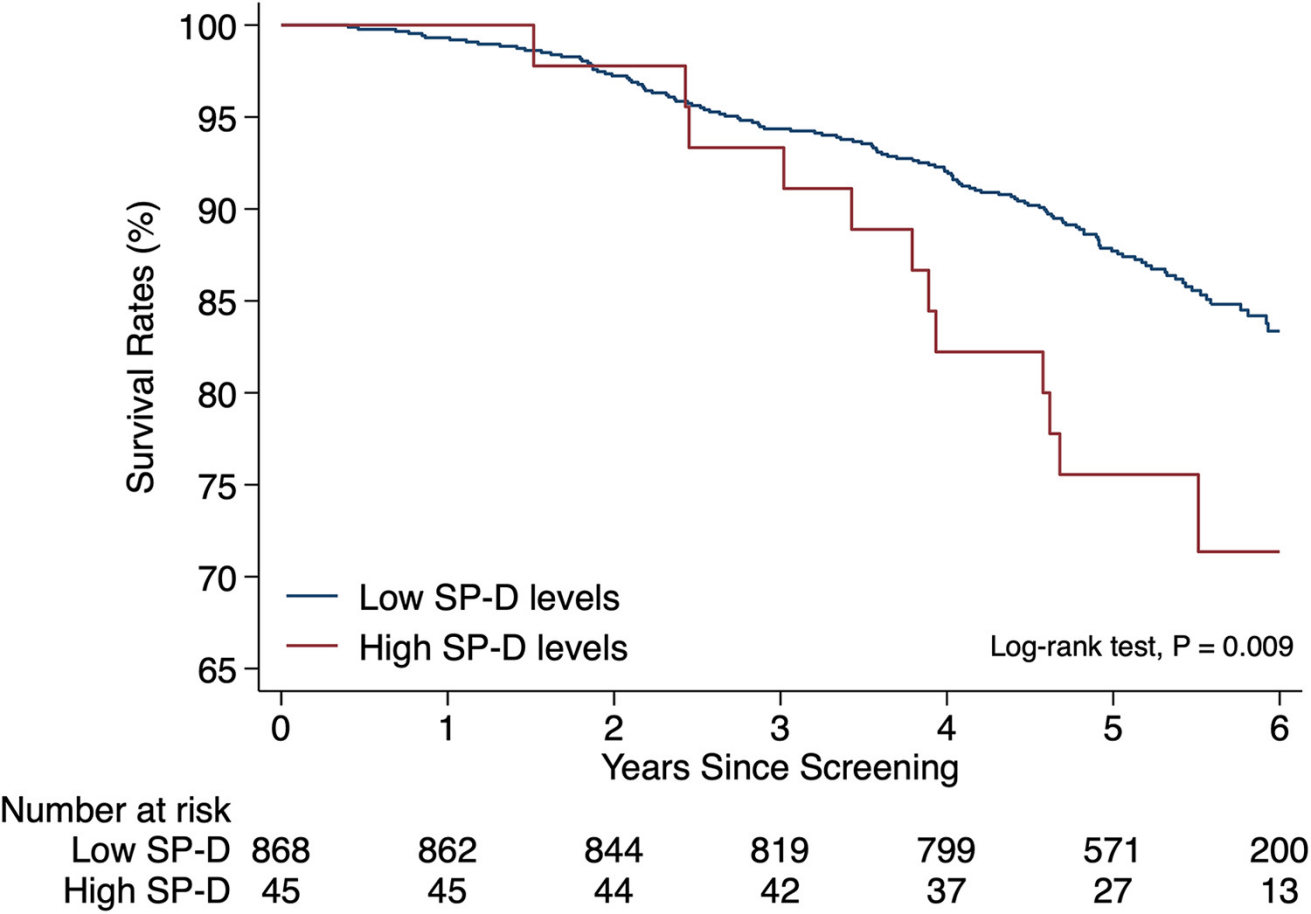
All-cause mortality in peripheral arterial disease patients with high vs low SP-D levels. Kaplan–Meier event plot of unadjusted cumulative proportions of deaths from all causes stratified by high (≥420.4 ng/ml) and low (<420.4 ng/ml) plasma levels of SP-D in patients with peripheral artery disease diagnosed by population-based screening. Patients were censored at the time of death or the end of follow-up. The difference in event rates between groups was tested with the log-rank test.

A significant difference in the crude hazard ratio (Table 2) and unadjusted cumulative proportions of deaths from all causes (Figure 2) was observed between the group of patients with high levels of SP-D compared with the group with lower levels. Additionally, hypertension, diabetes mellitus (DM), use of beta-agonists [an indicator of the presence of chronic obstructive pulmonary disease (COPD)], use of warfarin, and the degree of PAD were significantly associated with all-cause mortality (Table 2). When adjusting for those potential confounders, a strong and independent association persisted between high levels of SP-D and all-cause mortality with an adjusted hazard ratio of 2.40, 95% CI (1.36–4.24), *p* = 0.003, compared with the group with lower levels (Table 2). A satisfactory area under the ROC curve of 0.67 (95% CI: 0.62–0.72) was estimated when “high SP-D” was analyzed as a predictor of all-cause mortality in a multivariate analysis including the same confounders and time at risk.

**Table 2:** Univariate and multivariate Cox regression analysis of all-cause mortality in PAD patients.

Variables		Univariate analysis		Multivariate analysis		
All-cause mortality	Crude HR	95% CI	*p*-value	Adjusted HR*	95% CI	*p*-value
SP-D ≥420.4 ng/ml (95th percentile cutoff)	2.09	1.20–3.65	0.009	2.40	1.36–4.24	0.003
Age (per 1-year increase)	1.04	0.98–1.11	0.15	–	–	–
BMI	1.02	0.98–1.07	0.23	–	–	–
Hypertension	2.03	1.12–3.67	0.019	1.93	1.06–3.54	0.032
DM	2.10	1.46–3.01	<0.001	2.17	1.47–3.19	<0.001
Never smoker	REF	REF	REF	REF	REF	REF
Current smoker	1.92 1.36	1.02–3.63	0.044	2.00	1.05–3.81	0.036
Former smoker		0.71–2.58	0.36	1.10	0.57–2.14	0.77
Previous COPD	1.29	0.57–2.88	0.54	–	–	–
Previous CVD	1.05	0.71–1.54	0.82	–	–	–
Statin	1.04	0.74–1.46	0.81	–	–	–
Beta-agonists	2.71	1.19–6.15	0.017	4.02	1.72–9.38	0.001
Warfarin	1.70	1.04–2.79	0.034	1.76	1.05–2.95	0.031
PAD degree 1	REF	REF	REF	REF	REF	REF
PAD degree 2	1.73	1.13–2.64	0.012	1.52	0.98–2.36	0.06
PAD degree 3	2.09	1.31–3.34	0.002	1.61	0.98–2.63	0.05

Variables with an *α* level of <0.10 from univariate testing were included in the multivariate analysis. *Mutually adjusted for hypertension, diabetes mellitus, smoking status, use of beta-agonists and warfarin, and degree of peripheral arterial disease.

BMI, body mass index; CVD, cardiovascular disease; DM, diabetes mellitus; PAD, peripheral artery disease; SP-D, surfactant protein-D.

## 4 Discussion

Associations between SP-D and adverse events in PAD have been previously demonstrated and might also be expected due to observations of SP-D synthesis in vascular smooth muscle cells and endothelial cells (8, 9, 29), reduced atherosclerosis formation with SP-D ablation in mice (9, 23, 30), and the association between genetic polymorphisms affecting SP-D levels and atherosclerotic intima–media thickness (22). Here, we set out to test potential associations between SP-D and PAD in a new and larger cohort. We observed that plasma SP-D level is strongly predictive of all-cause mortality in PAD. The cause of this relation remains unknown.

We found no significant independent association between high SP-D and PAD degree or the risk of MACE, MALE, and MACLE. A previous study by Otaki et al. (24) showed that SP-D was an independent predictor of MACE and MACLE in a cohort of 364 PAD patients admitted to their first PAD treatment. An important difference between the study by Otaki et al. and the present study is that the former study included patients with advanced disease having endovascular therapy performed whereas our study included patients found through screening. Another difference was that our study only covered male data, whereas the cohort applied by Otaki et al. included 21% women. Complementary analyses on cohorts including female information are therefore urgently required. The combined male and female data existing in the previous cohort could suggest that female circulatory SP-D levels may be responsible for the prior observation of the significance of the link between SP-D and cardiovascular disease.

Moreover, of the patients enrolled in the study by Otaki et al., 41 patients were in the high SP-D (>110 ng/ml) group, corresponding to 11% of the total cohort. In our study, the high SP-D (>420.4 ng/ml) group consisted of 45 PAD patients, corresponding to 5% of the total cohort. We chose the 95th percentile cutoff to include those SP-D levels belonging to the right-skewed tail of the SP-D distribution. However, the definitions of high SP-D in Otaki et al. and this article are different. Additionally, the measurement methods of SP-D in the two studies are not the same, and it is well described that the choice of method can have a significant influence on the measured absolute concentration of SP-D (31). Importantly, studies including a limited number of subjects may lead to both false-positive and false-negative findings. The association between high SP-D levels and mortality has been shown in several studies in support of our conclusion. We cannot exclude that a larger cohort size would have allowed observation of the association between SP-D and MACE or MALE. However, we did not observe any such tendency.

Some of the covariates adjusted for in multivariate analysis are also different between the two studies. Otaki et al. adjusted for sex, ischemic heart disease, critical limb ischemia, and estimated glomerular filtration rate (eGFR), whereas the present study only included men, and associations were adjusted for previous CVD of any kind, hypertension, diabetes mellitus, use of beta-agonists and warfarin, medications associated with high risk of MACE/MACLE, and the degree of PAD.

Lack of information on some concomitant diseases might influence our observed SP-D levels, including the lack of adjustment during follow-up for ongoing infections, changes in pharmacological treatment and/or revascularizations, and the development of cancer in the multivariable models. However, our observed association between high SP-D levels and mortality is in line with other previous studies using populations both with and without selection for cardiovascular disease (13, 20). The observed association with all-cause mortality appears to be general and not restricted to cardiovascular disease or caused by cardiovascular disease in itself.

Of notice, in our cohort, we did not observe a higher prevalence of previously diagnosed chronic obstructive pulmonary disease (COPD) among individuals with elevated SP-D levels. However, the use of β-agonists during follow-up—often indicative of symptomatic respiratory disease—was strongly associated with all-cause mortality. Importantly, our analyses were adjusted for β-agonist use, and SP-D remained independently associated with mortality. Moreover, glucocorticoid treatment is indicative of respiratory disease. However, we initially excluded all patients receiving glucocorticoids due to the reported complex confounding effects of glucocorticoids on SP-D expression and circulatory levels (32, 33). These findings suggest that while respiratory disease may partially mediate the observed relationship, SP-D might also capture additional or alternative pathophysiological processes contributing to mortality risk.

Based on the above considerations, we suggest that future studies that could help settle the cause of the association between circulatory SP-D and mortality should include information on causes of death, importantly including cancer, such as lung cancer, stroke, and heart failure. Moreover, such a study should include the confounder’s sex (both sexes), age, cardiovascular disease severity, presence and type of diabetes, lung function measurements and respiratory disease diagnoses, the impact of current smoking such as exhaled carbon monoxide, measurement of renal function, measurement of overweight/obesity, and disease-relevant medications.

A weakness of our study may be the presence of lead-time bias. The screening-diagnosed PAD includes asymptomatic cases. Such early diagnosis of PAD due to identification by screening may have led to an apparent increase in survival time without necessarily improving overall outcomes. This bias occurs because patients are diagnosed earlier and thus have a longer period of observation. In our study, a relatively high survival rate above 95% was observed in the first 2 years of observation, and this may reflect such bias. In comparison, a 2-year survival rate of <80% was previously observed in a study of more advanced PAD patients who received percutaneous intervention (34). As a consequence of the relatively high 2-year survival rate in our study, “low SP-D” and “high SP-D” patients did not differ in survival rates in this early observation period.

The resulting area under the ROC curve suggested that “high SP-D” appears with satisfactory capacity as a biomarker of all-cause mortality. Other weaknesses include that we only studied SP-D biomarker capacity in a single cohort and that the nature of all-cause mortality was not available in this study.

As described above, a limitation was that we only investigated men. In addition, SP-D is known to undergo posttranslational modifications that change structure and function (35, 36). Importantly, SP-D appears to elicit different functions depending on the degree of multimerization (35, 37). The degree of multimerization is recognized to be affected by the presence of COPD, asthma (38), and axial spondyloarthritis (39). However, the degree of multimerization was not investigated in the present study.

In summary, we found that the plasma SP-D level is strongly predictive of all-cause mortality independently of established risk factors. SP-D thereby has the potential to serve as a mortality marker in addition to well-established clinical markers. No association between SP-D and PAD degree, MACE, or MALE was observed in men with screening-diagnosed PAD.

## Data Availability

The datasets presented in this article are not readily available because it is illegal to publish raw data from the National Danish Patient Registry. Requests to access the datasets should be directed to jes.sanddal.lindholt@rsyd.dk.
