# Supplementary material for: Surfactant protein-D is an independent predictor of all-cause mortality in men with peripheral artery disease diagnosed by population-based screening
Source: Front Cardiovasc Med. 2025 May 20;12:1534779. doi: 10.3389/fcvm.2025.1534779 (PMC12129913; doi:10.3389/fcvm.2025.1534779)
Supplement: Supplementary file 1 [file Datasheet1.pdf]

Supplementary Figure Legend

Supplementary Figure S1. Mean of the right and left Ankle-Brachial Index (ABI) obtained at time of screening in the Viborg Vascular Screening Trial (VIVA). Mean of the right and left Ankle-Brachial Index (ABI) obtained at time of screening in the Viborg Vascular Screening Trial (VIVA). PAD was defined as ABI below 0.9 or higher than 1.4, and the presented ABIs in the histogram are based on mean ABI values from both right and left measurements.

Supplementary Tables

Supplementary Table S1. Comorbidities, major adverse outcomes and causes of death according to registry data

Variable	Source	Codes
**Admission to hospital**		
Myocardial Infarction	ICD-10	I21-3
Apoplexy or TIA	ICD-10	I63-7
Ischemic heart disease ex. acute myocardial infarction	ICD-10	I20
Intracerebral Hemorrhage	ICD-10	I60-1
Peripheral occlusive arterial disease	ICD-10	I70 ex I701, I74 ex I742
**Surgical procedures**		
Percutaneous Angioplasty	NOMESCO/SKS	KFNG02, KFNG05
Coronary Artery Bypass Graft	NOMESCO/SKS	KFNA-E
Major Amputation	NOMESCO/SKS	KFNQ09, KFNQ19, KFNQ99, KNGQ09, KNGQ19, KNGQ99 (Variable value 1/16)
**Outcomes**		
Major Adverse Cardiac Events	Described above for each outcome included	Myocardial Infarction *OR* Percutaneous Angioplasty *OR* Coronary Artery Bypass Graft *OR* Stroke (Intracerebral Hemorrhage *OR* Apoplexy) *OR* Cardiovascular-related Death

Major Adverse Limb Events	Described above for each outcome included	Major Amputation *OR* Re-vascularization in Extremities *OR* Acute Ischemia in the Lower Extremities
Death-MALE-MACE	Described above for each outcome included	Death-MALE-MACE
**Causes of Death**		*Underlying Causes (Primary)*<br>*Contributory Causes (Secondary I-III)*
All Cause		
Cardiovascular Death	Described above for each outcome included	Myocardial Infarction, Ischemic heart disease, Intracerebral Hemorrhage, or Apoplexy

Inpatient and outpatient admissions are recorded in the National Patient Registry and classified according to the International Classification of Diseases, 10th Edition (ICD-10). The primary diagnosis is established at the time of discharge and signifies the main reason for hospitalization. This classification is utilized to identify key activities of interest based on ICD-10 codes.

Major vascular surgeries are registered in the national clinical database National Vascular Registry. The database includes a variable defining the indication for the procedure (F55, previously Indik1). That variable is used for identification of key procedures.

Coronary artery disease repair are identified in the National Patient Registry where all surgical procedures are characterized by the NOMESCO classification of surgical procedures (NOMESCO codes).

TIA (transient ischemic attack).

Supplementary Table S2. SP-D level distribution by PAD degree. Symptomatic PAD (claudication) was defined as pain while walking that subsided immediately upon stopping. Patients without these symptoms were categorized as having non-symptomatic PAD. PAD degree 1 (0.75<ABI<0.89 or 1.40<ABI<1.60), PAD degree 2 (0.5<ABI<0.75 or 1.6<ABI<1.8), and PAD degree 3 (ABI<0.5 or >1.8). Data are presented as medians (IQR: 25–75). The rank-sum test was used to compare overall SP-D levels between symptomatic and non-symptomatic PAD patients, while the Kruskal-Wallis test was applied to assess differences in SP-D levels across PAD severity categories. P-values <0.05 were considered significant. Abbreviations: Peripheral Arterial Disease (PAD), ABI (ankle brachial index), Surfactant Protein D (SP-D).

**Groups**	**Total N**	**Symptomatic PAD (n=325)**	**Non-Symptomatic PAD (n=578)**	***p*-value**
**Overall SP-D, ng/mL**	903	142 (100-201)	140 (95-212)	0.60
**SP-D level by PAD Degree, ng/mL**	903			
SP-D in PAD Degree 1	310	129 (90-190)	147 (98-222)	
SP-D in PAD Degree 2	403	152 (100-219)	134 (91-196)	0.59
SP-D in PAD Degree 3	190	147 (104-239)	139 (102-229)	
