# Supplementary figures and images for: Surfactant protein-D is an independent predictor of all-cause mortality in men with peripheral artery disease diagnosed by population-based screening
Source: Front Cardiovasc Med. 2025 May 20;12:1534779. doi: 10.3389/fcvm.2025.1534779 (PMC12129913; doi:10.3389/fcvm.2025.1534779)

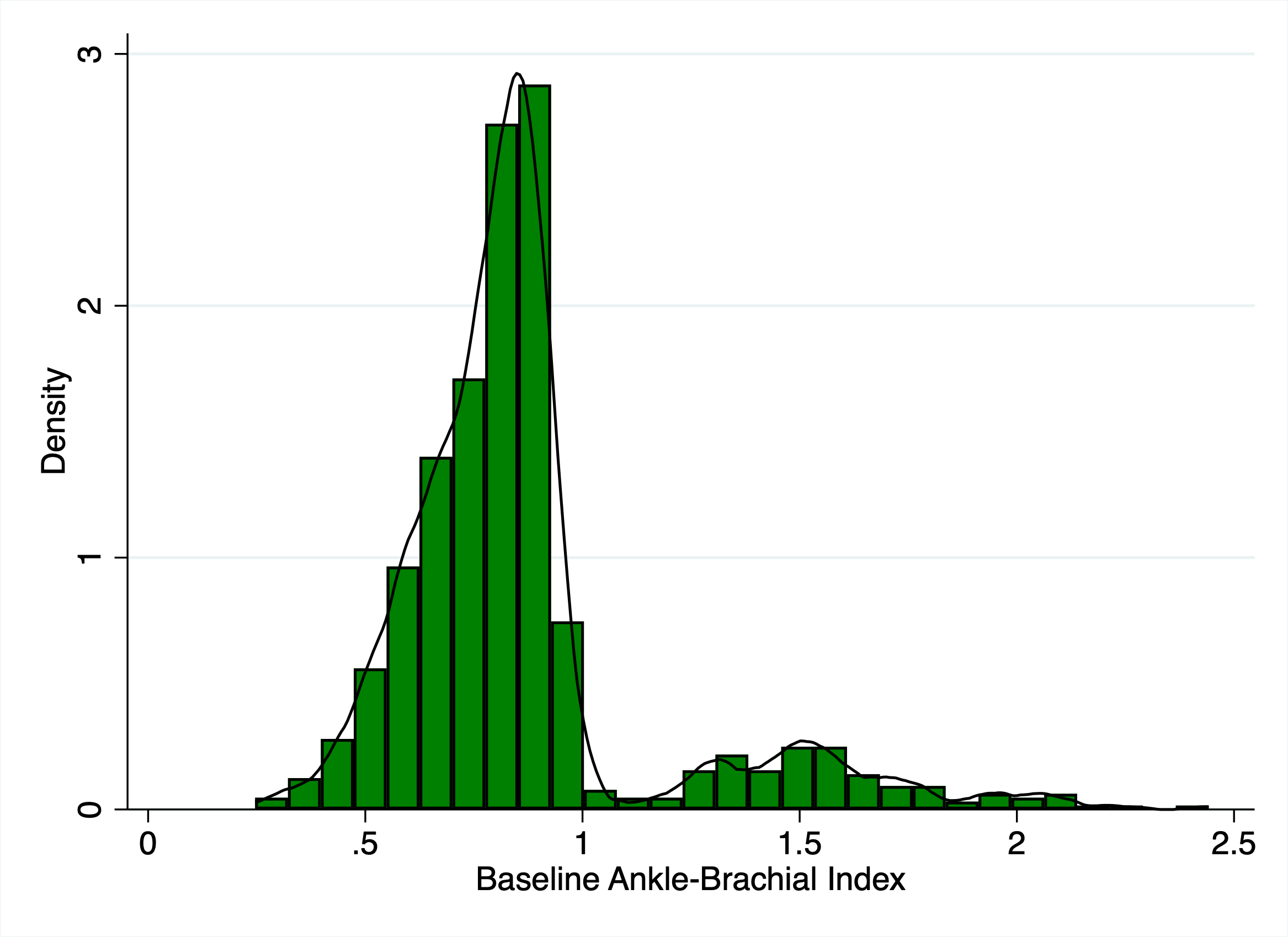

Supplement: Supplementary file 2 [file Image1.tif]
